# Recovery kinetics following sprint training: resisted versus unresisted sprints

**DOI:** 10.1007/s00421-023-05317-x

**Published:** 2023-09-30

**Authors:** Christina A. Liakou, Ioannis G. Fatouros, Athanasios Poulios, Themistoklis Tsatalas, Evangeli Karampina, Panagiota Karanika, Despoina Kaloudi, Anastasia Rosvoglou, Panagiotis Tsimeas, Anna Kamperi, Niki Syrou, Athanasios Gatsas, Konstantinos Papanikolaou, Dimitrios Draganidis, Panagiotis Tsaklis, Giannis Giakas, Athanasios Z. Jamurtas, Chariklia K. Deli

**Affiliations:** Department of Physical Education and Sport Science, School of Physical Education, Sport Science and Dietetics, University of Thessaly, Karies, 42100 Trikala, Greece

**Keywords:** Speed, Power, Exercise-induced muscle injury, Performance, Metabolism

## Abstract

**Purpose:**

To determine the recovery kinetics of performance and exercise-induced muscle damage following different sprint-training protocols.

**Methods:**

In a crossover design, ten male and female athletes (20.6 ± 2.4 years) performed 2 × (3 × 20 m: 2 min rest) and 1× (3 × 30 m: 3 min rest) of: (a) unresisted sprints (UST), (b) resisted sprints with 10% of body mass (BM) load (RST10), (c) resisted sprints with 20% BM load (RST20), against a control trial (no-training).

**Results:**

Blood lactate (mmol/L) increased post-training versus pre-training in all sprint-training trials (6.7 ± 2.4 vs 1.2 ± 0.2, 5.6 ± 2.4 vs 1.3 ± 0.3, 7.3 ± 2.7 vs 1.2 ± 0.3, in UST, RST10, RST20, respectively), as did creatine kinase (U/L) 24 h, 48 h and 72 h post-training (UST: 251 ± 173, 238 ± 154, 209 ± 115 vs 155 ± 9, RST10: 252 ± 134, 240 ± 83, 218 ± 103 vs 164 ± 106; RST20: 237 ± 133, 323 ± 303, 262 ± 184 vs 179 ± 106, respectively). DOMS of knee-extensors (KE) and knee-flexors (KF) increased post-training up to 72 h in all sprint-training trials versus pre-training (ranging from 1.6 ± 1.3 to 3.8 ± 2.8 vs 1.0 ± 0, respectively). Eccentric torque (N m) of the KE of the non-dominant limb, decreased 24 h post-training versus pre-training in all sprint-training trials (UST: 249 ± 49 vs 266 ± 54; RST10: 229 ± 52 vs 273 ± 72; RST20: 253 ± 6 vs 262 ± 56), as did that of the KF of the dominant limb (UST: 135 ± 29 vs 144 ± 26; RST10: 130 ± 29 vs 140 ± 25; RST20: 139 ± 33 vs 142 ± 26). 10-m sprint-time (s) increased 48 h post-training versus pre-training (1.81 ± 0.15 vs 1.77 ± 0.11), and 30-m sprint-time increased 24 h, 48 h, 72 h post-training versus pre-training (4.35 ± 0.36, 4.40 ± 0.44, 4.33 ± 0.41 vs 4.21 ± 0.34, respectively), only in RST20.

**Conclusions:**

Unresisted and resisted sprint-training induces prolonged reduction of muscle strength (24 h), and sprinting performance (72 h), associated with prolonged increase of DOMS and CK (72 h).

## Introduction

Sprinting requires an athlete to move forward with maximum velocity, and is pivotal for performance in numerous sport activities. Effective sprinting gives a critical advantage to both individual and team sports. For example, long-jumpers with an effective acceleration in run-up may have a greater potential for a longer jump (Bridgett and Linthorne 2006). Similarly, sprinting activities are very frequent in team sports as in soccer and rugby in all field positions (Haugen et al. 2014; Jeffs 2014), and linear sprinting usually precedes goal-scoring situations during a match (Faude et al. 2012; Jeffs 2014). Chronic sprint-training improves acceleration, maximal speed and power (Alcaraz et al. 2018; Rumpf et al. 2016; Sinclair et al. 2021). Thus, sprint-training represents one of the main components of a training program for many athletes.

Sprinting consists of distinct phases, i.e. the start, acceleration, maximum speed, and deceleration (Haugen et al. 2019). Several training methods have been developed to improve these phases and subsequently the overall sprinting performance (Alcaraz et al. 2018; Haugen et al. 2019, 2014; Jeffs 2014; Rumpf et al. 2016). Specifically, for acceleration training, several sprint distances are recommended (10–50 m) depending on the sport characteristics but also on the athletes’ training level, using both unresisted and resisted sprinting (Haugen et al. 2019; Jeffs 2014; Rumpf et al. 2016). Unresisted sprinting incorporates weight-bearing sprints with flying or static starts, whereas resisted sprinting includes uphill sprints, sled sprints or sprints with motorized devices (Haugen et al. 2019). Sled sprinting comprise one of the most examined methods consisting of linear sprints whilst towing a sled device with an additional load, prescribed either as a percentage of body mass (BM), a targeted reduction in velocity compared to unresisted sprint or as an absolute load, according to the sport’s demands or the daily training goals (Rumpf et al. 2016). Thus, for track and field sprinters resisting sprinting usually incorporates loads that decrease unresisted sprint velocity by 10–12%, whereas in other sports where athletes overcome external resistance, loads of 20–30% BM are used (Alcaraz et al. 2018). However, loads that decrease sprint velocity by even ~ 50% corresponding to 69–96% BM, have also been suggested as optimal to improve horizontal force and power production during early sprint acceleration (Cross et al. 2017). Although both unresisted and resisted sprint-training improve sprint acceleration and maximal sprint velocity (Rumpf et al. 2016; Sinclair et al. 2021), the most effective method has yet to be established (Alcaraz et al. 2018; Petrakos et al. 2016; Zabaloy et al. 2023).

Sprint-training frequency may affect performance adaptations (Ross and Leveritt 2001), and adequate recovery between sessions is a prerequisite for optimal training. Repeated unresisted maximal sprints may lead to metabolic fatigue post-training evidenced by increased lactate levels and decreased sprinting and jumping performance (Jimenez-Reyes et al. 2016). Additionally, sprinting utilizes the stretch–shortening cycle which incorporates an eccentric component known to provoke exercise-induced muscle damage (EIMD) (Fatouros and Jamurtas 2016), which induces local and systemic inflammation, muscle edema, increased leakage of muscle proteins into the circulation, delayed onset of muscle soreness (DOMS), and, eventually, reduction of muscle performance and function (Fatouros and Jamurtas 2016). EIMD is mainly produced as a result of strenuous and/or unaccustomed eccentric exercise (Deli et al. 2017a, b; Jamurtas et al. 2005; Margaritelis et al. 2021), thus, it could be argued that athletes that are familiarized with the eccentric component of sprint-training should not suffer from EIMD, yet, elevated DOMS and CK, and performance deterioration have been reported following sprint-related modalities in well-trained athletes (Johnston et al. 2015; Leeder et al. 2014; Tzatzakis et al. 2019). These reports indicate that EIMD may also occur in athletes even if they are familiarized with a specific training stimulus, and may compromise their performance during a subsequent training session or a competitive event the following days. Nevertheless, despite the utilization of acceleration sprint-training as a main component of the training program of many athletes, only limited scientific information exists regarding EIMD and the physiological responses and recovery the following days. Additionally, except for one study that used maximal velocity sprints (50 m) (Johnston et al. 2015), no other study so far examined the recovery beyond the 24 h following resisted acceleration sprint-training. Thus, the recovery kinetics of EIMD and performance the days following an acute linear acceleration sprint-training have not been adequately studied. Although usually sprint-training is repeated after at least 48 h, the design of microcycles is rather relying on empirical observations of coaches (Haugen et al. 2019), and not on scientific evidence. Additionally, most studies examined the related markers immediately post-training (Jimenez-Reyes et al. 2016, 2019). Thus, only few data exist on the effect of acute linear unresisted sprint-training the following days (Johnston et al. 2015; Thomas et al. 2018). As far as we know the only study that examined the effects of acute resisted linear acceleration sprinting beyond immediately post-exercise, employed a very limited recovery period of 24 h (Bachero-Mena et al. 2020). Scientific information regarding EIMD and the physiological responses and recovery following acceleration sprint-training over a multiple day timeframe is critical for practitioners to effectively design their training microcycles to reduce injury risk, and to maximize their athletes’ performance. Therefore, this investigation aimed to determine the recovery kinetics of EIMD, muscle function and performance markers, for up to 72 h following different linear acceleration sprint-training protocols, with or without additional external load in well-trained athletes.

## Methods

### Study design

**Fig. 1:**
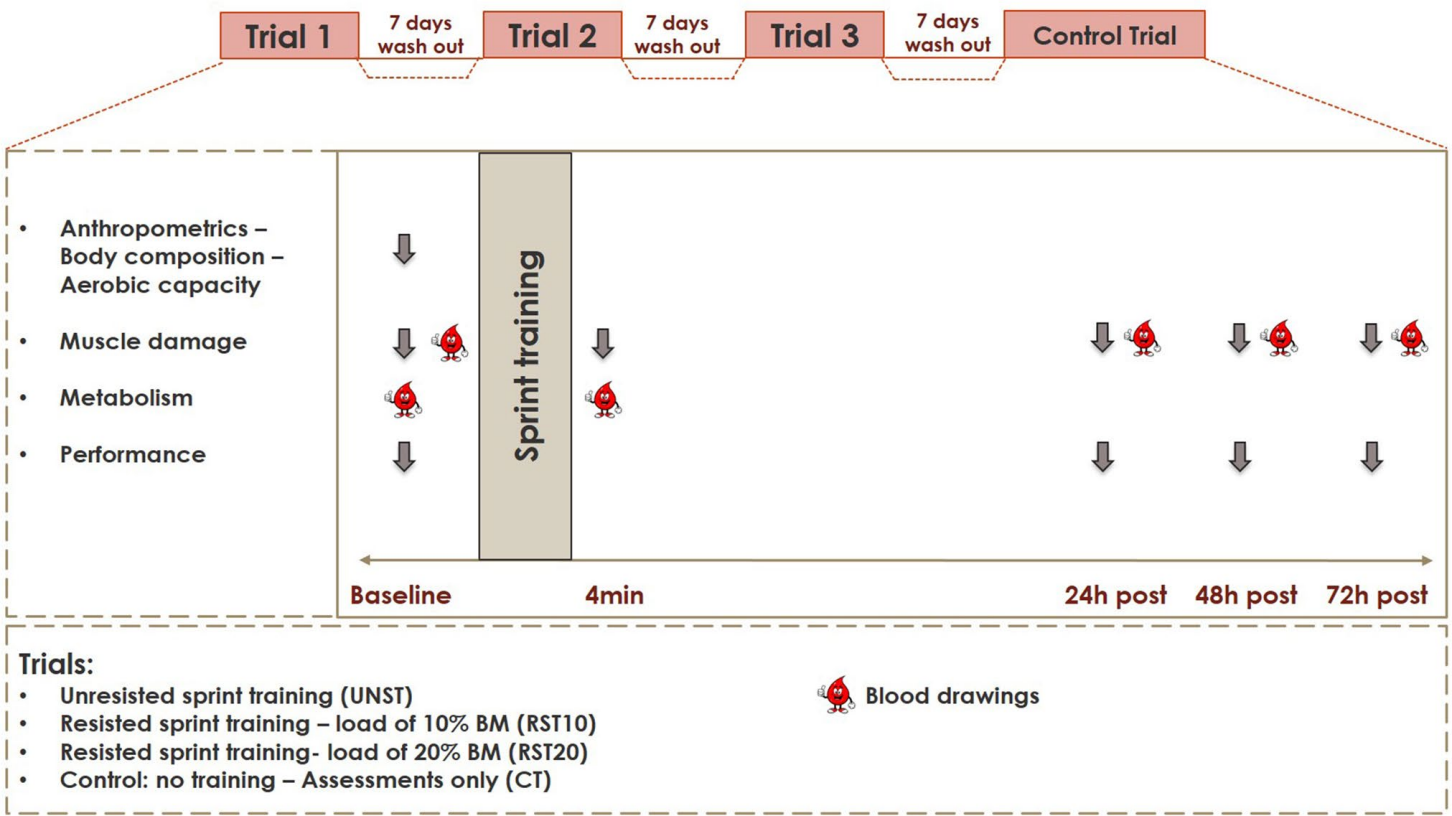
Study design

The study used a randomized, cross over, controlled, repeated measures design (Fig. 1). Participants signed an informed consent form after they were fully informed about all the benefits and risks of the study and filled in and signed a medical history questionnaire. Participants were then instructed by a dietitian how to record a 7-days diet recall to ensure that they would not consume to greater extent nutrients that may affect EIMD and fatigue (e.g. antioxidants, amino acids, etc.) and that the energy intake during the trials would be the same. Somatometric assessment and measurement of aerobic capacity (VO_2max_) were performed. Thereafter, participants randomly performed one of the four different trials: (a) unresisted sprint-training (UST), (b) resisted sprint-training with additional external load of 10% BM (RST10), (c) resisted sprint-training with additional external load of 20% BM (RST20), or (d) control trial (no training—measurements only, CT). The randomization of the trials was made via a random integer set generator (Random.org) available online.

Blood sampling (to measure CK), and assessment of 10-m and 30-m sprint-time, 10-m and 30-m average speed, DOMS of knee extensors (KE) and flexors (KF) of dominant (DL) and non-dominant (NDL) limb, concentric and eccentric isokinetic peak torque, and countermovement jump (CMJ), were performed pre-, and at 24, 48 and 72 h post-exercise. DOMS was further assessed post-exercise. Blood lactate concentration [La], was determined pre-exercise and four minutes post-exercise to measure the peak post-exercise concentration (Fujitsuka et al. 1982). The participants performed the remaining trials, according to the previous randomization. Between trials, a 7-day wash out period was adapted.

### Participants and ethics statement

**Fig. 2:**
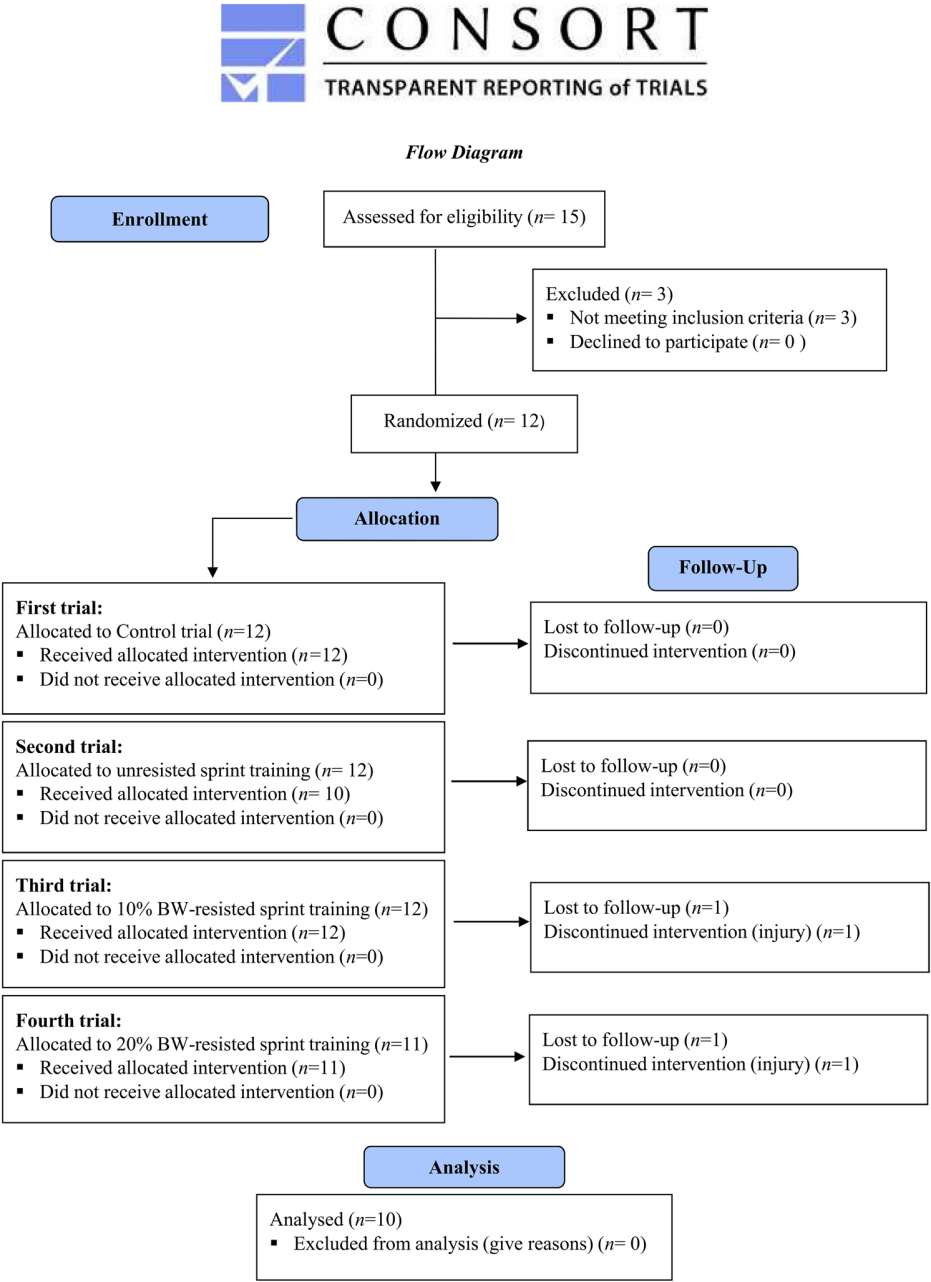
Consort flow diagram

According to a preliminary power analysis (a probability error of 0.05 and a statistical power of 80%), a sample size of 8–10 participants/group was considered appropriate to detect statistically meaningful changes between trials. Thus, ten male and female competitive athletes (sprinters, long jumpers, and soccer players) aged 18–26 years participated in the study (Fig. 2). Sprinters and long jumpers were competing in the National championships, while soccer players were competing at first division level. Participants were training 5–6 days/week, at least 2–3 h/day, performing acceleration sprint-training twice per week, and were all familiarized both with unresisted and sled-pulling sprint-training. Participants’ baseline characteristics are shown in Table 1. Volunteers were eligible to participate in the study if: (a) were competitive athletes incorporating sprinting in their regular training, (b) had no musculoskeletal injury during the last 6 months, (c) abstained from use of ergogenic supplements, medications and/or drugs for the last 2 months, (d) abstained from eccentric exercise training for at least 7 days before the study, (e) abstained from alcohol or energy drink consumption before the study. All participants provided written informed consent to participate to the study, following detailed explanation of the benefits and potential risks. Procedures were in accordance with the 1964 Declaration of Helsinki, as revised in 2013 and approval was received from the Ethics Committee of the School of Physical Education and Sport Science of the University of Thessaly (Protocol number: 1715/09-12-2020). The study is registered at ClinicalTrials.gov (Identifier: NCT04766411).

**Table 1:** Baseline characteristics of the participants

Participants	1	2	3	4	5	6	7	8	9	10	Mean (SD)
Age (years)	26	18.1	20.6	18.8	21.0	18.5	21.8	18.9	19.7	22.7	20.6 (2.4)
Body mass (kg)	79.7	69.17	65.8	64.46	77.8	51.1	69.0	76.3	69.7	62.6	68.6 (8.4)
Height (m)	1.80	1.83	1.76	1.77	1.82	1.58	1.82	1.70	1.82	1.77	1.77 (0.1)
BMI (kg/m^2^)	24.5	20.6	21.7	20.5	23.8	21.1	21.3	26.4	20.5	20.0	22.0 (2.1)
Body fat (%)	14.5	9.1	13.5	13.3	14.8	20.8	18.1	42.3	13.0	19.8	17.9 (9.2)
Fat mass (kg)	11.1	6.2	8.4	8.4	11.0	9.3	11.9	29.3	8.2	11.6	11.5 (6.5)
Lean mass (kg)	65.0	60.1	53.8	54.7	63.5	34.0	53.8	37.9	52.8	47.0	52.3 (10.2)
Fat free mass (kg)	68.7	63.0	57.5	58.1	66.8	36.0	57.1	40.8	55.7	49.6	55.3 (10.6)
BMC (g)	3658	2890	3659	3349	3367	1978	3276	2913	2843	2682	3062 (511)
BMD (g/cm^2^)	1.40	1.35	1.42	1.35	1.32	1.30	1.27	1.35	1.31	1.24	1.33 (0.06)
VO_2max_ (ml/kg/min)	52.9	54.5	51.9	54.5	55.4	57.2	47.7	45.6	54.4	46.9	51.89 (4.9)
10-m sprint time (s)	1.65	1.89	1.70	1.71	1.64	1.89	1.89	1.86	1.58	1.79	1.78 (0.1)
30-m sprint time (s)	3.88	4.01	3.96	4.02	3.91	4.48	4.61	4.89	3.91	4.26	4.19 (0.4)
CMJ height (cm)	47.7	41.3	33.1	39.0	42.8	30.6	28.7	18.6	35.0	30.4	34.72 (8.4)

*BMI* Body mass index, *BMC* Bone mineral content, *BMD* Bone mineral density, *VO*_*2max*_ Maximal oxygen consumption, *CMJ* Countermovement jump

### Dietary analysis

The participants recorded their dietary intake (Table 2) for seven days before each trial to ensure that the athletes diet was consistent with the typical suggestions regarding daily macronutrients intake (i.e., 20–30% of total energy for fat, 3–10 g kg^−1^ BM for carbohydrates, and 1.2–2 g kg^−1^ BM for protein) for optimal performance during training (Markus et al. 2021; Thomas et al. 2016). Participants received a copy of their dietary record sheets, and were asked to follow the same diet before the other trials so that the energy intake and the consumption of nutrients that may affect EIMD and fatigue (e.g. antioxidants, amino acids, etc.) (Markus et al. 2021) would be the same during the trials to avoid a potential effect of diet on the recovery process. Dietary records were analyzed using the ScienceFit Diet 200A (Science Technologies, Athens, Greece).

**Table 2:** Participants’ dietary analysis

	Mean (SD)
Daily energy intake (kJ)	8976 (2809)
Daily energy intake (kcal)	2153 (680)
Daily protein intake (% of total energy content)/(g kg^−1^ BM)	21.2 (3.53)/1.3 (0.5)
Daily carbohydrate intake (% of total energy content)/(g kg^−1^ BM)	52.8 (7.52)/3.1 (0.8)
Daily fat intake (% of total energy content)	25.9 (6.51)
Vitamin E (mg)	9.6 (4.8)
Vitamin C (mg)	89 (39.8)

*BM* Body mass

### Exercise trials and control trial

Each training protocol consisted of 2 sets of 3 × 20 m sprints and 1 set of 3 × 30 m sprints. 2-min and 3-min rest periods were applied between repetitions for the 20-m and 30-m sprints, respectively, while a 3-min rest was applied between sets. The specific training protocol was selected according to current practice and sprint training recommendations for acceleration improvement in athletics and team sports (Haugen et al. 2019; Jeffs 2014; Petrakos et al. 2016). The same indoor track and field stadium and sled model (Amila, Vws-1835, India) (for resisted sprinting) were used to avoid variations in the coefficient of friction of the running surface and ultimately an inconsistency in training load (Linthorne and Cooper 2013). All the athletes performed the sprints wearing spikes. Each trial (including the control trial) was performed in a random order in order to minimize the repeated bout effect.

### Blood sampling and assays

After an overnight fast, blood samples (8 mL) were collected from an antecubital arm vein by venipuncture using a disposable 10-gauge needle with the participants sitting as previously described (Deli et al. 2017a, b). Blood was collected into tubes containing coagulation agent and after remaining at room temperature for 20 min to clot, it was centrifuged (1370*g*, 10 min, 4 °C) and serum was collected. Serum was stored in multiple aliquots at − 80 °C and thawed only once before the analysis of CK.

### Assessment of somatometrics and aerobic capacity

Body mass was measured to the nearest 0.05 kg (Beam Balance 710; Seca, Birmingham, United Kingdom) while being lightly dressed and barefoot, and standing height was measured to the nearest 0.1 cm (Stadiometer 208; Seca, Birmingham, United Kingdom) as previously described (Deli et al. 2017a, b). Body mass index was calculated as mass per height squared. Body composition (fat mass, fat-free mass, fat percent, lean body mass, bone mineral density, bone mineral content) was assessed by dual emission X-ray absorptiometry (DXA, GE-Healthcare, Lunar DPX NT, Belgium) as described before (Tzatzakis et al. 2019).

Μaximal oxygen consumption (VO_2max_) was assessed through an automated online pulmonary gas analyzer (Vmax Encore 29, BEBJO296, Yorba Linda, CA, USA) during a graded exercise protocol on a treadmill (Stex 8025T, Korea). Participants performed a warm-up of ~ 8 min on the treadmill (~ 60% of maximal heart rate) and ~ 5 min of stretching exercises. The initial speed for the testing protocol was 10 km h^−1^, then increased at 11 km h^−1^ for the second minute, and increased thereafter by 0.5 km h^−1^ for every one minute until exhaustion. The O_2_ and CO_2_ rates in the exhaled air were measured through a gas analyzer (Vmax Encore 29, BEBJO296, Yorba Linda, CA, USA), which was calibrated before each test using commercially available precision gases (16% O_2_, 4% CO_2_, 80% N_2_), while the heart rate was recorded continuously using a heart rate monitor (Polar Tester S610TM, Electro Oy, Finland). VO_2max_ was determined if at least three of the following criteria were fulfilled: (i) a < 2 mL kg^−1^ min^−1^ increase in VO_2_ with an increase in work rate, (ii) respiratory quotient > 1.10, (iii) heart rate within 10 bpm of the theoretical maximum heart rate (Gellish et al. 2007), (iv) participant’s exhaustion.

### Lactate

[La] was assessed in capillary blood (~ 0.7 μL) collected through puncturing of the middle finger with a sterile needle, using a hand portable analyzer (Lactate Plus, Nova Biomedical, USA) as previously described (Tzatzakis et al. 2019). Blood lactate was measured four minutes post-exercise to determine the peak post-exercise concentration (Fujitsuka et al. 1982).

### Muscle damage

DOMS of KE and KF of both limbs was evaluated. The participants performed three repetitions of full-squats, and rated their soreness on a visual analog scale from 1 to 10 (with “no pain” at one end and “extremely sore” at the other) using palpation of the belly and the distal region of relaxed KE and KF. CK was measured using an automated Clinical Chemistry Analyzer (HumanStar 200, HUMAN, Magdeburg, Germany) with commercially available kits (HUMAN, Magdeburg, Germany).

### Performance and muscle function

Sprint-time (sec) at 10-m and 30-m sprint was measured on an indoor track and field stadium with participants wearing spikes, using infrared photocells with a precision of 0.01 s (Newtest, Finland) as previously described (Tzatzakis et al. 2019), and the best of the two attempts was recorded. Average speed during the 10-m and 30-m sprint was calculated by dividing sprint-time by the distance covered.

During a CMJ test, jump height, ground reaction force, peak power, mean power, vertical stiffness, and peak rate of force development, were measured on two force platforms at 1000 Hz (Bertec, FP4060‐10‐2000, Bertec Corporation, Columbus, OH, USA), with each foot in parallel on the two platforms providing a separate yet time‐synchronized measurement data for each leg as previously described (Mina et al. 2019).

Isokinetic strength was assessed through maximal concentric and eccentric torque of the KE and KF muscles of both limbs on an isokinetic dynamometer (Cybex, HUMAC NORM 360, Ronkonkoma, NY) at 60° s^−1^ as previously described (Deli et al. 2017a, b).

### Statistical analysis

The normality of the data distribution was examined with a Shapiro–Wilk test. CK and DOMS were not normally distributed and were analyzed with non-parametric tests. Friedman and Wilcoxon Signed Rand tests were performed for within trials analysis and Kruskal–Wallis and Mann–Whitney tests were applied for between trials comparisons. All the remaining dependent variables were analyzed using a two-way ANOVA (trial × time) with repeated measures on time to examine possible differences on recovery, and between trials differences. Statistical significance was set at p < 0.05. Effect Sizes (ESs) and confidence intervals (CIs) were calculated using the Hedge g method, corrected for bias. Accordingly, ES was interpreted as trivial, small, medium-sized, and large for values 0.00–0.19, 0.20–0.49, 0.50–0.79, and ≥ 0.8, respectively. Statistical analyses were performed with SPSS, version PASW 18.0 (SPSS Inc., Chicago, Ill.). The results are presented as mean ± SD.

## Results

No differences were noted at baseline measurements before each trial, indicating that the wash-out period was effective to eliminate any inflammatory effects from a previous trial. No changes from baseline were noted in any of the estimated indices in the CT throughout the study.

### Lactate

**Fig. 3:**
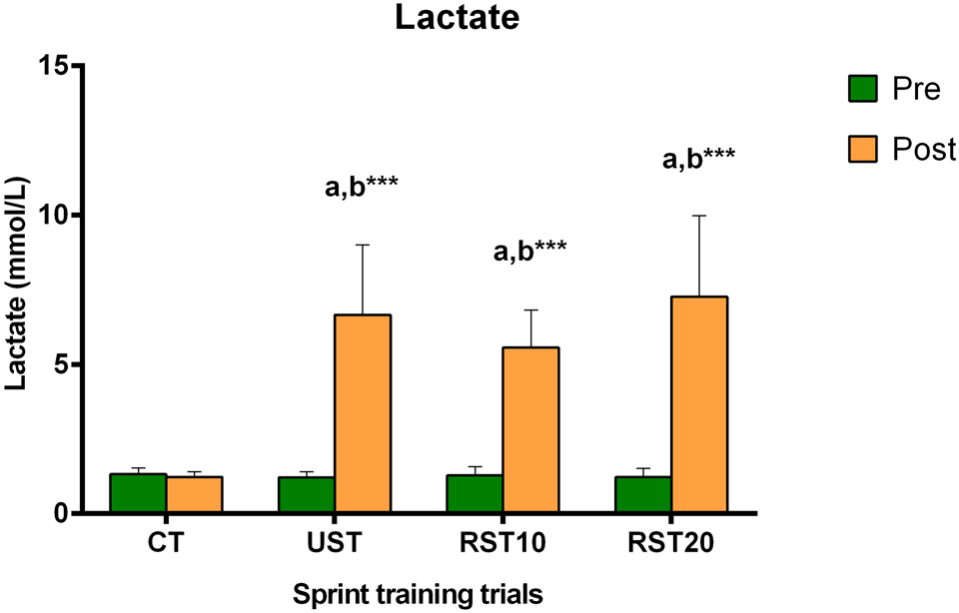
Changes in [La] during recovery following control trial (CT) and sprint training with unresisted sprints (UST), resisted sprints with 10% BM (RST10) and 20% BM (RST20) external load. ^a^Different compared to pre-training. ^b^Different compared to CT. ***p < 0.001

Changes in [La] are presented in Fig. 3. [La] increased post-training compared to pre-training (p < 0.001) in all sprint-training trials (6.7 ± 2.4 vs 1.2 ± 0.2, 5.6 ± 2.4 vs 1.3 ± 0.3, 7.3 ± 2.7 vs 1.2 ± 0.3 mmol L^−1^, in UST, RST10, RST20, respectively).

The corresponding ES and CI for the above changes were: UST (ES = − 3.13, CI = − 4.43 to − 1.82); RST10 (ES = − 4.46, CI = − 6.10 to − 2.82); and RST20 (ES = − 2.98, CI = − 4.25 to − 1.71).

The [La] rise was comparable among sprint-training trials.

### Muscle damage

#### Creatine kinase

**Fig. 4:**
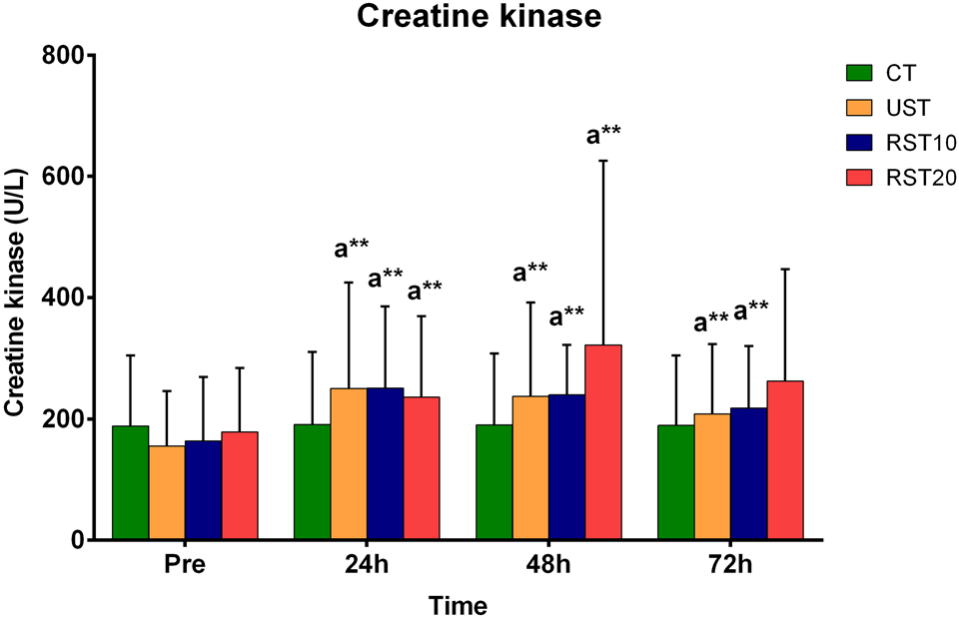
Changes in creatine kinase during recovery following control trial (CT) and sprint training with unresisted sprints (UST), resisted sprints with 10% BM (RST10) and 20% BM (RST20) external load. ^a^Different compared to pre-training in the same trial. **p < 0.001

Changes in CK activity are presented in Fig. 4. CK increased 24 h, 48 h and 72 h post-training compared to pre-training in UST and RST10 trials [UST: 251 ± 173, 238 ± 154, 209 ± 115 vs 155 ± 9 U/L, at 24 h (p = 0.009), 48 h (p = 0.022), 72 h (p = 0.009), respectively; RST10: 252 ± 134, 240 ± 83, 218 ± 103 vs 164 ± 106 U/L, at 24 h (p = 0.005), 48 h (p = 0.017), 72 h (p = 0.00), respectively], while at 24 h (p = 0.016) and 48 h (p = 0.017) post-training compared to pre-training in RST20 trial [237 ± 133, 323 ± 303 vs 179 ± 106 U/L, respectively).

The corresponding ES and CI for the above changes were: UST (24 h: ES = − 0.66, CI = − 1.56 to 0.24, 48 h: ES = − 0.62, CI = − 1.52 to 0.28, 72 h: ES = − 0.49, CI = − 1.38 to 0.40); RST10 (24 h: ES = − 0.70, CI = − 1.60 to 0.21, 48 h: ES = − 0.77, CI = − 1.68 to 0.14, 72 h: ES = − 0.50, CI = − 1.39 to 0.39); RST20 (24 h: ES = − 0.46, CI: − 1.35 to 0.43, 48 h: ES = − 0.61, CI: − 1.50 to 0.29).

CK responses were comparable among sprint-training trials.

### Delayed onset of muscle soreness

Changes in DOMS are presented in Table 3. In the DL, DOMS of the KE and KF increased in UST at 24 h and 48 h compared to pre-training (p < 0.05), in RST10 increased post-, 24 h, and 48 h compared to post-training (p < 0.05). In RST20, DOMS of the KE increased 48 h (p < 0.05), and of KF post- and 72 h post-training (p < 0.05) compared to pre-training.

**Table 3:** Changes in DOMS during recovery

	Baseline	Post	24 h	48 h	72 h
Knee extensors of the dominant limb					
Control trial	1 (0.0)	1 (0.0)	1 (0.0)	1 (0.0)	1 (0.0)
Unresisted trial	1 (0.0)	1.6 (1.3)	2.9 (2.3)^a^*^,b^* (− 1.10; − 2.05 to − 0.16)	2.7 (1.9)^a^*^,b^* (− 1.22; − 2.17 to − 0.26)	2.1 (1.9)
10% resisted trial	1 (0.0)	2.8 (1.6)a*,b* (− 1.57; − 2.58 to − 0.57)	3.8 (2.8)^a^*^,b^** (− 1.34; − 2.32 to − 0.37)	3.6 (2.6)^a^*^,b^* (− 1.38; − 2.36 to − 0.41)	2.6 (2.2)^b^*^,c^*
20% resisted trial	1 (0.0)	1.9 (1.5)	2.8 (2.6)	2.2 (1.6)^a^*^,b^* (− 1.05; − 1.98 to − 0.11)	1.6 (1.3)
Knee extensors of the non-dominant limb					
Control trial	1 (0.0)	1.0 (0.0)	1.0 (0.0)	1.0 (0.0)	1.0 (0.67)
Unresisted trial	1 (0.0)	1.9 (1.5)	3.5 (2.1)^a^*^,b^** (− 1.60; − 2.60 to − 0.59)	2.2 (1.6)^a^*^,b^* (− 1.05; − 1.98 to − 0.11)	1.3 (1.00)
10% resisted trial	1 (0.0)	2.2 (1.6)^b^*	4.2 (2.4)^a^**^,b^*** (− 1.84; − 2.89 to − 0.80)	3.7 (2.1)^a^*^,b^** (− 1.78; − 2.81 to − 0.74)	2.9 (2.1)^a^*^,b^*^,c^* (− 1.21; − 2.16 to − 0.26)
20% resisted trial	1 (0.0)	2.2 (1.6)^a^*^,b^* (− 1.05; − 1.98 to − 0.11)	2.6 (2.2)^b^*	3.3 (1.70)^a^*^,b^** (− 1.83; − 2.88 to − 0.79)	1.9 (1.5)
Knee flexors of the dominant limb					
Control trial	1 (0.0)	1 (0.0)	1 (0.0)	1 (0.0)	1 (0.0)
Unresisted trial	1 (0.0)	1.6 (1.3)	4.0 (3.2)^a^*^,b^* (− 1.27; − 2.23 to − 0.31)	2.9 (2.1)^a^*^,b^* (− 1.21; − 2.16 to − 0.26)	2.1 (1.9)
10% resisted trial	1 (0.0)	2.7 (1.9)^a^*^,b^* (− 1.21; − 2.17 to − 0.26)	3.3 (2.7)^a^*^,b^* (− 1.17; − 2.11 to − 0.22)	3.6 (2.7)^a^*^,b^* (− 1.29; − 2.26 to − 0.33)	2.2 (1.6)
20% resisted trial	1 (0.0)	2.5 (1.6)^a^*^,b^* (− 1.29; − 2.25 to − 0.32)	2.8 (2.6)	2.4 (1.9)^b^*	2.2 (1.6)^a^* (− 1.05; − 1.98 to − 0.11)
Knee flexors of the non-dominant limb					
Control trial	1 (0.0)	1 (0.0)	1 (0.0)	1 (0.0)	1.0 (0.0)
Unresisted trial	1.0 (0.0)	1.6 (1.3)	3.3 (2.8)^a^* (− 1.10; − 2.04 to − 0.16)	2.9 (2.1)^a^* (− 1.21; − 2.16 to − 0.26)	2.1 (1.9)
10% resisted trial	1.0 (0.0)	2.2 (1.6) ^a^* (− 1.05; − 1.98 to − 0.11)	3.6 (2.6)^a^* (− 1.38; − 2.36 to − 0.41)	3.6 (2.4)^a^* (− 1.49; − 2.48 to − 0.50)	2.4 (1.9)^a^* (− 1.00; − 1.93 to − 0.07)
20% resisted trial	1.0 (0.0)	2.5 (1.6)^a^* (− 1.29; − 2.25 to − 0.32)	2.9 (2.1)^a^* (− 1.21; − 2.16 to − 0.26)	2.7 (1.9)^a^* (− 1.21; − 2.17 to − 0.26)	2.5 (1.6)^a^* (− 1.29; − 2.25 to − 0.32)

Changes are presented as mean (SD); for significant differences, ES and CI are also presented

*DOMS* Delayed onset of muscle soreness, *KE* Knee extensors, *KF* Knee flexors

*p < 0.05; **p < 0.01; ***p < 0.001

^a^Different from baseline

^b^Different from control trial

^c^Different from unresisted trial

In the NDL, DOMS of the KE and KF increased in UST at 24 h and 48 h post-training compared to pre-training (p < 0.05), and so did in RST10 throughout recovery (p < 0.05). In RST20, DOMS of the KE increased post- and 48 h post-training compared to pre-training (p < 0.05), and so did DOMS of the KF throughout recovery (p < 0.05).

Compared to CT, DOMS rise of the KE and KF was greater throughout recovery in all sprint-training trials, with some exceptions.

DOMS rise was comparable among sprint-training trials throughout recovery, except for 72 h, where DOMS of KE was greater in RST10 compared to UST for both limbs.

The exact p values for each time-point, as well as the corresponding ES and CI for the above changes are presented in Table 3.

### Performance

#### Sprint performance

**Fig. 5:**
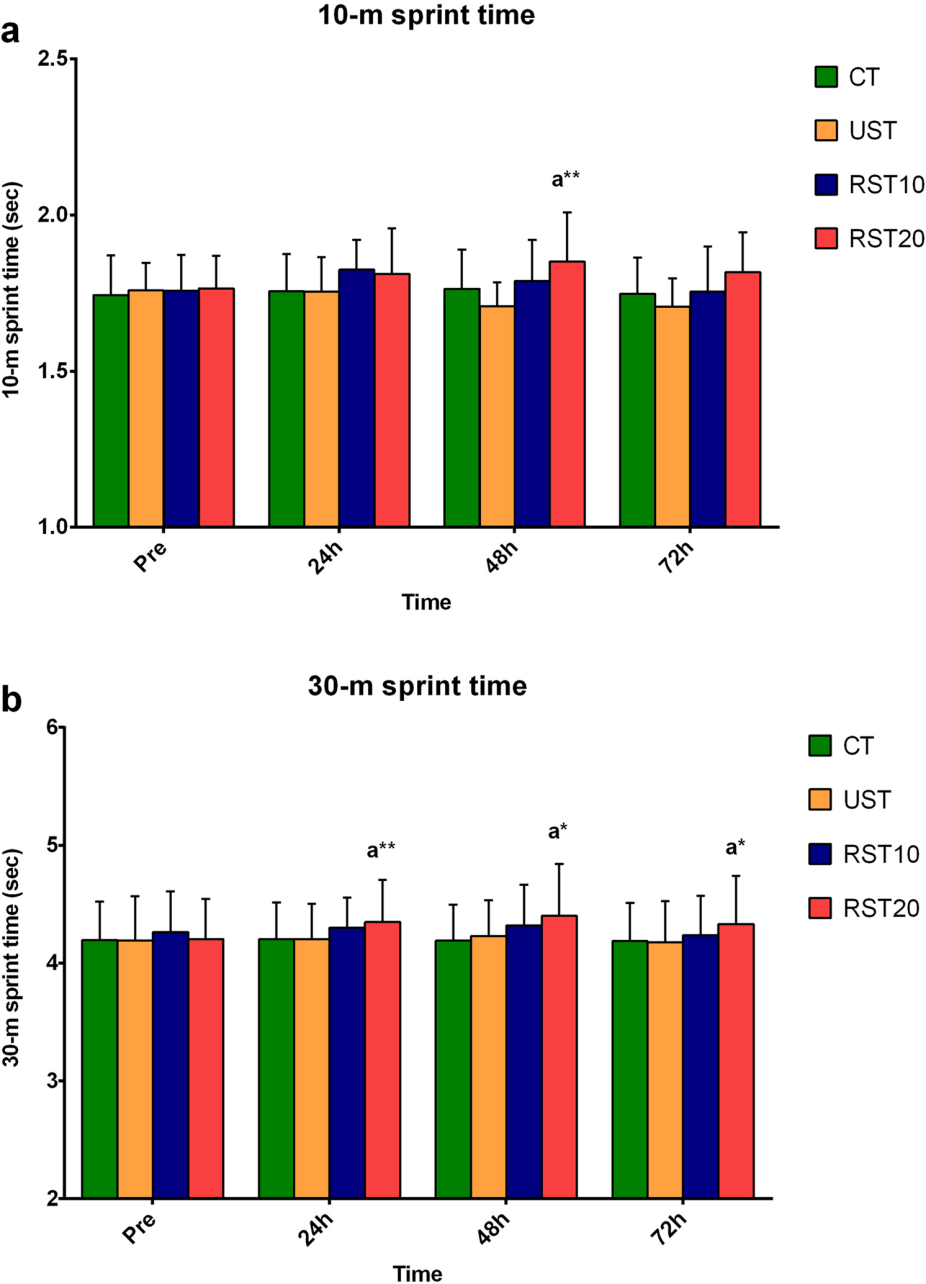
Changes in 10 m-sprint time (**a**) and 30 m-sprint time (**b**) during recovery following control trial (CT), sprint training with unresisted sprints (UST), resisted sprints with 10% BM (RST10), and resisted sprints with 20% BM (RST20) external load. Different compared to pre-training at the same trial. *p < 0.05. **p < 0.01

Changes in sprint performance are presented in Fig. 5. 10-m sprint-time increased 48 h (p = 0.056) post-training compared to pre-training (1.851 ± 0.16 vs 1.765 ± 0.11 s), and 10-m average speed decreased 48 h (p = 0.056) post-training versus pre-training (19.581 ± 1.67 vs 20.462 ± 1.20 km h^−1^), only in response to RST20.

30-m sprint-time increased 24 h (p = 0.012), 48 h (p = 0.001), and 72 h (p = 0.054) post-training compared to pre-training (4.349 ± 0.36, 4.402 ± 0.44, 4.333 ± 0.41 vs 4.205 ± 0.34 s, respectively), and 30-m average speed decreased 24 h (p = 0.038) and 48 h (p = 0.003) post-training versus pre-training (25.223 ± 1.92, 24.912 ± 2.36 vs 25.829 ± 1.97 km h^−1^, respectively), only in response to RST20.

The corresponding ES and CI for the above changes were: 10-m sprint-time (ES = − 0.62, CI = − 1.52 to 0.28); 10-m average speed (ES = − 0.59, CI = − 0.31 to 4.48); 30-m sprint-time (24 h: ES = − 0.39, CI: − 1.28 to 0.49; 48 h: ES = − 0.55, CI: − 1.44 to 0.35; 72 h: ES = − 0.33, CI: − 1.21 to 0.56); 30-m average speed (24 h: ES = − 0.30, CI: − 1.58 to 1.18; 48 h: ES = 0.40, CI: − 0.48 to 1.29).

Sprint performance changes were comparable among sprint-training trials.

### Isokinetic strength

Changes in isokinetic strength are presented in Table 4. The isokinetic strength of the KE of the DL, as well as of the KF of the NDL did not change from pre-training throughout recovery. Eccentric peak torque of the KE in the NDL (p = 0.039) decreased in all sprint-training trails 24 h post-training compared to pre-training (KE-NDL: 249 ± 49 vs 266 ± 54, 229 ± 52 vs 273 ± 72, 253 ± 61 vs 262 ± 56 N∙m, in UST, RST10, RST20, respectively), and so did eccentric peak torque of the KF of the DL (p = 0.041) (135 ± 29 vs 266,144 ± 26, 130 ± 29 vs 140 ± 25, 139 ± 33 vs 142 ± 25 N m, in UST, RST10, RST20, respectively).

**Table 4:** Changes in isokinetic strength during recovery

	Baseline	24 h	48 h	72 h
Knee extensors, concentric peak torque of the dominant limb (N m)				
Control	191 (36)	186 (39)	197 (39)	187 (30)
Unresisted	195 (38)	201 (38)	192 (26)	191 (35)
10% BM Resisted	199 (38)	195 (39)	198 (53)	198 (41)
20% BM Resisted	187 (36)	189 (45)	191 (41)	187 (36)
Knee extensors, concentric peak torque of the non-dominant limb (N m)				
Control	191 (39)	187 (37)	195 (44)	187 (38)
Unresisted	200 (41)	197 (34)	197 (39)	194 (36)
10% BM resisted	200 (46)	198 (37)	190 (39)	202 (50)
20% BM resisted	195 (49)	198 (52)	183 (51)	186 (32)
Knee flexors, concentric peak torque of the dominant limb (N m)				
Control	117 (21)	113 (21)	119 (23)	117 (17)
Unresisted	115 (20)	115 (23)	119 (16)	110 (21)
10% BM resisted	118 (19)	115 (22)	120 (18)	121 (26)
20% BM resisted	116 (20)	119 (26)	110 (16)	112 (15)
Knee flexors, concentric peak torque of the non-dominant limb (N m)				
Control	119 (23)	119 (21)	120 (24)	115 (21)
Unresisted	12 (29)	114 (20)	115 (23)	115 (14)
10% BM resisted	123 (28)	120 (21)	114 (23)	119 (26)
20% BM resisted	119 (28)	120 (29)	108 (22)	116 (20)
Knee extensors, eccentric peak torque of the dominant limb (N m)				
Control	254 (40)	246 (45)	249 (49)	241 (45)
Unresisted	269 (62)	257 (56)	259 (50)	246 (63)
10% BM resisted	274 (61)	256 (49)	260 (87)	254 (62)
20% BM resisted	251 (45)	259 (52)	244 (36)	251 (43)
Knee extensors, eccentric peak torque of the non-dominant limb (N m)				
Control	247 (43)	245 (41)	244 (48)	242 (49)
Unresisted	266 (54)	249 (49)^a^* (0.31; − 0.57 to 1.20)	251 (57)	246 (61)
10% BM resisted	273 (72)	229 (52)^a^* (0.67; − 0.23 to 1.57)	249 (70)	249 (56)
20% BM resisted	262 (56)	253 (61)^a^* (0.14; − 0.74 to 1.01)	237 (54)	251 (48)
Knee flexors, eccentric peak torque of the dominant limb (N m)				
Control	147 (29)	141 (29)	146 (34)	134 (30)
Unresisted	144 (26)	135 (29)^a^* (0.29; − 0.60 to 1.17)	138 (25)	137 (28)
10% BM Resisted	140 (25)	130 (29)^a^* (0.35; − 0.54 to 1.23)	140 (32)	133 (30)
20% BM Resisted	142 (25)	139 (33)^a^* (0.10; − 0.78 to 0.98)	133 (25)	133 (25)
Knee flexors, eccentric peak torque of the non-dominant limb (N m)				
Control	147 (32)	150 (32)	147 (32)	144 (32)
Unresisted	148 (37)	141 (25)	142 (30)	142 (30)
10% BM resisted	150 (37)	144 (31)	152 (28)	148 (34)
20% BM resisted	151 (32)	155 (41)	139 (44)	148 (36)

Values are presented as mean (standard deviation, SD); for significant differences, ES and CI are also presented

*p < 0.05

^a^Different from baseline

Isokinetic strength of the KE and KF of both limbs were comparable between training trials throughout the study.

The corresponding ES and CI for the above changes are presented in Table 4.

### Countermovement jump

Changes in CMJ are presented in Table 5. All CMJ parameters were not significantly affected by all trials throughout recovery.

**Table 5:** Changes in countermovement jump performance during recovery

	Baseline	24 h	48 h	72 h
Jump height (m)				
Control trial	0.342 (0.07)	0.336 (0.06)	0.341 (0.07)	0.335 (0.06)
Unresisted	0.354 (0.07)	0.344 (0.07)	0.334 (0.07)	0.336 (0.08)
10% resisted	0.355 (0.08)	0.351 (0.07)	0.350 (0.08)	0.346 (0.07)
20% resisted	0.346 (0.08)	0.351 (0.07)	0.350 (0.08)	0.349 (0.07)
Peak ground reaction force (N)				
Control trial	991 (210)	981 (179)	1003 (216)	1025 (187)
Unresisted	1010 (243)	1001 (266)	1004 (285)	1007 (247)
10% resisted	980 (188)	976 (199)	1008 (218)	1019 (254)
20% resisted	1018 (269)	1038 (274)	989 (243)	987 (277)
Peak power concentric (W)				
Control trial	51.7 (9.5)	51.5 (9.2)	51.9 (9.7)	51.5 (8.7)
Unresisted	53.6 (10.4)	52.8 (8.4)	51.6 (9.8)	51.8 (10.8)
10% resisted	54.0 (7.2)	52.2 (9.1)	53.1 (8.9)	53.7 (7.9)
20% resisted	52.5 (8.6)	52.5 (10.4)	52.7 (9.6)	53.0 (10.1)
Peak power eccentric (W)				
Control trial	− 18.6 (4.2)	− 18.0 (4.1)	− 19.2 (4.9)	− 18.0 (4.8)
Unresisted	− 18.1 (4.0)	− 17.0 (4.0)	− 17.4 (6.0)	− 18.5 (5.2)
10% resisted	− 17.4 (4.5)	− 18.0 (3.9)	− 18.0 (3.3)	− 17.4 (5.2)
20% resisted	− 18.9 (5.4)	− 20.8 (4.5)	− 18.6 (4.7)	− 19.5 (4.7)
Power mean concentric (W)				
Control trial	28.5 (5.1)	27.9 (4.4)	28.2 (5.0)	27.8 (4.7)
Unresisted	29.4 (5.5)	28.6 (3.9)	27.7 (5.7)	28.1 (5.9)
10% resisted	29.7 (3.6)	28.7 (5.1)	28.9 (4.3)	29.3 (4.3)
20% resisted	29.3 (4.7)	29.1 (5.9)	28.9 (4.9)	28.2 (5.2)
Power mean eccentric (W)				
Control trial	− 6.7 (0.9)	− 6.6 (0.6)	− 6.9 (0.8)	− 6.6 (1.0)
Unresisted	− 6.7 (0.8)	− 6.3 (1.1)	− 6.4 (1.2)	− 6.5 (1.0)
10% resisted	− 6.2 (1.4)	− 6.9 (0.9)	− 6.6 (1.0)	− 6.2 (1.7)
20% resisted	− 6.6 (1.4)	− 7.0 (0.9)	− 6.7 (1.0)	− 6.9 (0.8)
RSIMOD				
Control trial	0.488 (0.12)	0.475 (0.10)	0.482 (0.12)	0.462 (0.09)
Unresisted	0.502 (0.12)	0.475 (0.11)	0.460 (0.14)	0.469 (0.13)
10% resisted	0.480 (0.10)	0.481 (0.10)	0.489 (0.10)	0.479 (0.12)
20% resisted	0.479 (0.13)	0.501 (0.14)	0.480 (0.14)	0.494 (0.14)
Vertical stiffness (N m^−1^)				
Control trial	46.2 (10.1)	46.7 (9.5)	44.3 (11.5)	47.2 (10.7)
Unresisted	47.3 (13.0)	50.9 (18.3)	48.5 (19.5)	47.7 (15.0)
10% resisted	54.3 (19.9)	43.3 (10.1)	47.5 (13.7)	47.7 (14.1)
20% resisted	54.6 (22.0)	46.0 (11.0)	45.6 (11.1)	45.7 (12.1)

Values are presented as mean (standard deviation, SD)

## Discussion

This study, examined for the first time the recovery kinetics of muscle damage and performance following unresisted and resisted (using a load of 10% and 20% BM) acceleration sprint-training protocols over a 72 h period. The main findings were that (i) acute unresisted and resisted acceleration sprint-training increase [La] similarly, (ii) acute unresisted and resisted acceleration sprint-training induce EIMD in well-trained athletes, familiarized to sprint-training, which may persist for 72 h, (iii) sprint performance is deteriorated during recovery only following resisted sprints with 20% BM, (iv) eccentric peak force of KE and KF may be compromised for 24 h following unresisted and resisted acceleration sprint-training, and (v) CMJ performance is not affected following acceleration sprint-training with these characteristics.

[La] usually rises during and after acute high-intensity exercise due to the upregulated muscle glycolysis to cover the increased energy demands (Spriet 1992). The magnitude of [La] rise depends on exercise intensity, with higher intensities resulting in higher [La] (Fiorenza et al. 2019), which may exceed 20 mmol L^−1^ following intense exercise (Osnes and Hermansen 1972). Energy demands during a short maximal sprint (≤ 10 s), are mainly derived from the breakdown of stored muscle phosphagens (i.e. adenosine triphosphate, phosphocreatine), and glycolysis (Bogdanis et al. 1998). Repeated short sprints during the same training session increases markedly the [La] (Bogdanis et al. 1998). Elevated [La] is accompanied by high levels of H^+^ and pH decrease, which to some degree, may contribute to fatigue and muscle function impairment (Allen et al. 2008). Here, sprint training consisting of maximum linear sprints of 20-m and 30-m, under external loading of either 0%, 10% or 20% BM, increased similarly [La]. Although not statistically meaningful, it must be mentioned that RST20 provoked the highest rise (six-fold), followed by UST (5.5-fold), and RST10 (4.3-fold) trial. Similar [La] responses have been reported for external loads up to 20% BM while higher loads (60% and 80% BM) elicit even a greater [La] rise (Bachero-Mena et al. 2020), i.e. linear sprint training may induce a load-dependent lactate elevation. It also appears that fatigue progressively increases with heavier external loads during acceleration training (Bachero-Mena et al. 2020). A potential limitation of this investigation is that fatigue index during the training sessions and performance immediately post-training were not evaluated and thus an association between training-induced fatigue and [La] responses is not possible. The magnitude of [La] elevation following linear sprint-acceleration training seems to be lower compared to other sprint-training modalities such as maximal speed training (16.5 mmol L^−1^) (Jiménez-Reyes et al. 2019), repeated sprint training (12.7 mmol L^−1^) (Gharbi et al. 2014), or speed endurance training (15.3 mmol L^−1^) (Tzatzakis et al. 2019). Differences in variables such as total distance covered, and training density may induce different metabolic demands that result in different lactate responses.

Exercise-induced muscle damage usually occurs as a result of strenuous and/or unaccustomed eccentric exercise (Deli et al. 2017a; b; Jamurtas et al. 2005; Margaritelis et al. 2021), thus it could be argued that athletes that are familiarized with sprint-training should not suffer from EIMD. However, EIMD symptoms have also been reported in well-trained athletes following sprint-related modalities (Johnston et al. 2015; Kritikos et al. 2021; Leeder et al. 2014; Tzatzakis et al. 2019). In this study, all the athletes were familiarized with unresisted and resisted sprint-training, yet, EIMD occurred following training as evidenced by the rise in CK and DOMS, and the deterioration of muscle strength and performance the subsequent days. These findings indicate that EIMD may also occur in well-trained athletes despite their familiarization with the training stimulus, which may compromise their performance during a subsequent training session or a competitive event the following days, and attention is needed. Weather EIMD symptoms the days following acceleration sprint-training are of greater magnitude and duration in non-familiarized individuals compared to well-trained familiarized athletes, needs to be investigated; to our knowledge there is no such data regarding acceleration sprint-training.

CK is a muscle protein, which, under situations of muscle trauma diffuses into the extracellular space due to destruction of the cytoplasmic membrane and increases in the circulation (Noakes 1987). CK usually peaks 24–48 h post-exercise, depending on the extend of muscle injury, and recovers several days after (Deli et al. 2017a; b; Jamurtas et al. 2005; Papanikolaou et al. 2021). In this study, CK increased following all exercise trials demonstrating its greatest rise (1.8-fold) in RST20 trial at 48 h, followed by UST (1.6-fold) and RST10 (1.5-fold) trials at 24 h. These findings agree with those reported by Bachero-Mena et al. (2020) who found CK responses of a similar magnitude 24 h post-training with loads up to 20% BM. It appears that CK elevation following acceleration sprint-training is of rather moderate magnitude, compared to those reported following other speed-related training modalities such as maximal speed (990 IU L^−1^) (Johnston et al. 2015), repeated-sprints (1320 IU L^−1^) (Leeder et al. 2014), speed endurance (458 IU L^−1^) (Tzatzakis et al. 2019), or plyometric (1700 IU L^−1^) (Huang et al. 2021) training protocols probably due to differences in total distance covered, training density, incorporated decelerations due to changes of direction and participants’ level. It is worth noting that CK presents high interindividual variability both at rest and when exposed to the same exercise protocol even when other non-modifiable factors such as gender, age, and training status are accounted for (Baird et al. 2012). High- and low-responders to exercise in respect to CK elevation have been reported in the literature (Brancaccio et al. 2007). Thus, the validity of CK as an EIMD marker may be questionable (Baird et al. 2012) although its monitoring after exercise may be valuable as a biomarker of prolonged fatigue and subclinical disorders (Baird et al. 2012; Brancaccio et al. 2007). Consistent with this phenomenon, interindividual variability was also evident in this study. Resting CK ranged from 84 to 426 ΙU L^−1^ probably due to inclusion of participants from both genders. Similar variability in CK values was also noticed the following days post-exercise among participants.

DOMS is a key-symptom of EIMD and usually increases after several hours and may persist for several days following exercise cessation (Deli et al. 2017a; b; Jamurtas et al. 2005; Papanikolaou et al. 2021; Tzatzakis et al. 2019). In this study, DOMS increased following all exercise trials in both KE and KF for both limbs. The magnitude of DOMS rise was similar between trials, although more prolonged (72 h) DOMS was observed under the RST10 trial for the KE, and under RST20 trial for the KF. Additionally, DOMS kinetics mimicked those of CK during recovery, i.e. peak changes were seen at 24–48 h post-training. As far as we know, no other studies estimated DOMS following acceleration sprint-training, thus no direct comparison can be made. Nevertheless, DOMS magnitude and recovery kinetics under both unresisted and resisted conditions were similar with that recorded following other sprint-related protocols (Johnston et al. 2015; Kritikos et al. 2021; Tzatzakis et al. 2019).

EIMD is also associated with prolonged deterioration of muscle strength and performance (Deli et al. 2017a; b; Draganidis et al. 2015; Fatouros and Jamurtas 2016; Papanikolaou et al. 2021; Tzatzakis et al. 2019). Prolonged (1–14 days) muscle strength reduction (10–70%) is considered as one of the most valid markers of EIMD since among all markers they exhibit the highest correlation with histological evidence of muscle disruption (Clarkson and Hubal 2002; Fatouros and Jamurtas 2016). In this study, eccentric peak torque of the KE of the NDL and KF of the DL declined up to 24 h post-exercise in all sprint-training trials, while sprint-performance was deteriorated the following days only after RST20 trial. Eccentric peak torque of the KF has been correlated with horizontal force production and electromyography activity during acceleration sprinting, and impairment of KF force is related with lower acceleration performance (Morin et al. 2015). Thus, we could speculate that the reduction of eccentric peak torque observed in this study may partially explain the deterioration of sprint performance, at least, following the RST20 trial. Others reported a decrement of concentric mean power of KF at 24 h post-training with an external load of 20% BΜ (Bachero-Mena et al. 2020). Considering that all types of muscle actions are incorporated in sprinting, a compromised eccentric or concentric function of either the KE or KF, may ultimately lead to deteriorated sprint performance during recovery.

The fact that sprint-performance was deteriorated the following days only after RST20 trial, suggests that external loads greater than 10% BM are needed to affect performance in both early- and late-acceleration phase. Although Bacchero-Mena et al. (2020) examined sprint performance for a brief period, it was evident that heavier external loads were associated with greater performance decline. In that study performance recovered earlier compared to this study, but training and testing included only 20-m sprints, while performance recovery was measured up to 24 h. Sprint-performance deterioration has also been reported following other speed-related training modalities (Tzatzakis et al. 2019).

Interestingly, CMJ height and its related parameters were not affected during recovery, regardless the reduction in sprint performance in RST20. Similar results have been reported by others using similar or heavier external loads (Bachero-Mena et al. 2020).

The deterioration of sprint performance, indicates that acceleration sprint-training also induces neuromuscular fatigue (Fiorenza et al. 2019; Tzatzakis et al. 2019), that impairs maximal power during sprint lasting for 72 h. Neuromuscular fatigue may be central-type associated to changes in neural drive, motor unit recruitment and/or firing frequency, or peripheral-type related to changes in muscles’ contractile properties and metabolic perturbations such as depleted energy substrates and accumulation of metabolic by-products (Fiorenza et al. 2019). Previous research on other sprint-related protocols report a more pronounced central fatigue (Tzatzakis et al. 2019), but also a combination of central and peripheral fatigue (Thomas et al. 2018). On the other hand, powerful actions like jumping critically depend on rapid muscle force generation by the KE, which has been reported to remain relatively unchanged under fatiguing conditions (Thorlund et al. 2009). Thus, it could be supported that the unaltered rapid force generation by the KE extensors, may partly explain the absence of CMJ performance deterioration the following days after acceleration sprint-training (Bachero-Mena et al. 2020), or the smaller and more short-lived deterioration of CMJ compared to sprinting performance following other sprint-related training modalities (Tzatzakis et al. 2019). However, in a previous study the CMJ height loss immediately post maximal speed-training, correlated with the observed velocity loss in 60-m running speed (Jiménez-Reyes et al. 2019). The controversial results among studies may be due to the different training protocols used. Jiménez-Reyes et al. focused on maximal speed using longer sprint distances (60 m) and total volume which have produced higher metabolic responses and neuromuscular fatigue compared to this, and other studies (Bachero-Mena et al. 2020). Additionally, contrary to this study, Jiménez-Reyes et al. (2019) examined CMJ performance during and immediately post-training, and not the following days. Thus, it cannot be supported with certainty that the observed loss in CMJ height, and its correlation with the velocity loss would also remain the following days. Additional research is needed to clarify the responses of CMJ height and its related parameters, and its association with velocity loss, the following days after acceleration sprint-training.

An issue that needs to be considered in studies with a cross over design regarding the recovery from exercise, is that the results may be influenced due to the repeated bout effect (Hyldahl et al. 2017). However, this is more likely to occur if the participants perform all the trials with the same order (Chen et al. 2020), and with unaccustomed exercise (Deli et al. 2017a; b; Jamurtas et al. 2005; Margaritelis et al. 2021). In this study, all of the participants were familiarized with resisted and unresisted sprint-training, and each participant performed each trial in a random order to minimize the repeated bout effect. The large standard deviation in CK at 48 h in the RST20 trial could mean that the repeated bout effect had blunted the CK activity. However, the phenomenon of high CK interindividual variability itself, could account for the large standard deviations observed in CK under the RST20 trial. Additionally, there are discrepant results regarding the CK response following a second bout of EIMD protocol. For example, after the second bout of sport-specific EIMD protocol, unaltered (Leeder et al. 2014), as well as greater (Chen et al. 2020) rise in CK has been reported, although DOMS attenuation was evident in both studies. Thus, CK may not be the most suitable index to determine a repeated bout effect and should be interpreted with caution. Nevertheless, the comparable response of CK, DOMS, and eccentric torque reduction between the training-trials and the deterioration of sprint-performance in RST20 trial, indicates that the recovery process in this study were not influenced by the repeated bout effect. Further research with appropriate design is needed to determine the possibility of the repeated bout effect phenomenon on recovery from repetitive acceleration sprint-training, in well-trained, familiarized athletes.

## Conclusions

Sprint-acceleration training with unresisted and resisted sprints reduces sprint performance and induces EIMD. Higher loads increase metabolic demands and induce greater performance decline and this should be considered by coaches during training programming to optimize athletic performance and minimize injury risk. Short sprint-accelerations and jumps may be repeated 48 h after unresisted and 10% BM-resisted sprint-training, and more than 72 h of recovery are needed after 20% BM-resisted sprint-training.

## Data Availability

The datasets generated and/or analyzed during the current study are available from the corresponding author on reasonable request.

## Abbreviations

ANOVA: Analysis of variance
BM: Body mass
CK: Creatine kinase
CMJ: Countermovement jump
CI: Confidence intervals
CT: Control trial
DL: Dominant limb
DOMS: Delayed onset of muscle soreness
ES: Effect size
EIMD: Exercise-induced muscle damage
KE: Knee extensors
KF: Knee flexors
[La]: Lactate concentration
NDL: Non-dominant limb
UST: Unresisted sprint-training
RST10: Resisted sprint-training with 10% of body mass
RST20: Resisted sprint-training with 20% of body mass
